# Post traumatic stress symptom variation associated with sleep characteristics

**DOI:** 10.1186/s12888-020-02550-y

**Published:** 2020-04-16

**Authors:** Quinn M. Biggs, Robert J. Ursano, Jing Wang, Gary H. Wynn, Russell B. Carr, Carol S. Fullerton

**Affiliations:** 1 Center for the Study of Traumatic Stress (CSTS), Department of Psychiatry, Uniformed Services University of the Health Sciences (USUHS), 4301 Jones Bridge Road, Bethesda, MD 20814 USA; 2 Walter Reed National Military Medical Center (WRNMMC), Bethesda, MD USA

**Keywords:** Post-traumatic stress disorder, Sleep, Symptom assessment, Ecological momentary assessment, Military personnel

## Abstract

**Background:**

Post traumatic stress disorder (PTSD) and sleep problems are highly related. The relationship between nighttime sleep characteristics and next day post traumatic stress symptoms (PTSS) is not well known. This study examined the relationship between the previous night’s sleep duration, number of awakenings, sleep quality, trouble falling asleep, and difficulty staying asleep and PTSS the following day.

**Methods:**

Using an ecological momentary assessment methodology, individuals with probable PTSD (*N* = 61) reported their nighttime sleep characteristics daily and PTSS four times per day for 15 days. Univariate and multivariate linear mixed models were used to examine the previous night’s (within-subjects) and person’s mean (between-subjects) associations between sleep characteristics and PTSS.

**Results:**

The previous night’s sleep duration (*p* < .001), sleep quality (*p* < .001), trouble falling asleep (*p* < .001), and difficulty staying asleep (*p* < .001) significantly predicted the next day’s PTSS. When examined in a multivariate model including all characteristics simultaneously, previous night’s sleep duration (*p* = .024), trouble falling asleep (*p* = .019), and difficulty staying asleep (*p* < .001) continued to predict PTSS, but sleep quality (*p* = .667) did not. When considering a person’s mean, trouble falling asleep (*p* = .006) and difficulty staying asleep (*p* = .001) predicted PTSS, but only difficulty staying asleep (*p* = .018) predicted PTSS in a multivariate model.

**Conclusions:**

Among individuals with PTSD, the previous night’s sleep duration, trouble falling asleep, and difficulty staying asleep predict next day PTSD symptoms. Interventions that facilitate falling and staying asleep and increase time slept may be important for treating PTSD.

## Background

Post traumatic stress disorder (PTSD) and sleep problems are highly related [1]. The presence of sleep disturbance in individuals with PTSD has been found to range from 70 to 92% in general and military population samples [2–4]. Insomnia (difficulty falling or staying asleep or restless sleep) and recurring nightmares are part of the diagnosis of PTSD [5] and are among the most frequently reported post traumatic stress symptoms (PTSS) [6, 7]. Individuals with PTSD are more likely to have a shorter sleep duration [8], have more nighttime awakenings [9], and report sleep quality as poor [10] compared to those without PTSD.

Although disturbed sleep has been considered a secondary symptom of PTSD, several lines of evidence suggest that it is an independent risk factor for PTSD [1, 11]. Sleep disturbance that is present prior to exposure to a traumatic event [12–14] or after exposure to a traumatic event [15] predicts the development of PTSD. Among those with PTSD, increased sleep disturbance is associated with increased PTSS severity [16]. Sleep disturbance often remains after successful treatment for PTSD [7, 17, 18] and greater residual sleep disturbance is predictive of smaller PTSD treatment gains [19]. Conversely, pharmacologic treatments for sleep [20–22] and non-pharmacologic treatments for sleep [23–25] improve sleep and PTSS.

Sleep disturbance and PTSD may feedback upon one another to exacerbate and maintain both conditions [1, 11, 26, 27]. For example, healthy sleep may facilitate recovery from PTSD through the consolidation of fear extinction memories [28]. However, ongoing sleep disturbance may interfere with consolidation of memories and perpetuate PTSD [9, 29].

While there is a clear association between sleep disturbance and PTSD, few studies have examined the day-to-day temporal relationship between them. Sleep and PTSS are often assessed using global retrospective measures (i.e., assessing sleep or PTSS over the past month). Such assessment methods may not capture daily variability or the impact that a good or bad night’s sleep might have on PTSS. Ecological momentary assessment (EMA), a method of repeated experience sampling of subjects in their natural environment, is well-suited to assessing change in symptoms over time [30, 31]. Recent studies have used EMAs to examine PTSS from day to day [32–35], sleep diaries to examine sleep disturbance from night to night [8, 36], and EMAs and sleep diaries combined to examine the temporal relationship between sleep disturbance and PTSS [37–39].

Short et al. [38] examined the relationship between the previous night’s sleep disturbance (i.e., sleep duration, efficiency, quality, and nightmares) and the next day’s PTSS in a community and undergraduate sample with PTSD (*N* = 30; 61.3% female) and found, after accounting for the prior evening’s PTSS, reduced sleep efficiency and poor sleep quality (but not sleep duration or nightmares) predicted increased PTSS and negative affect the next day. In another study that used the same sample but changed the direction of the analyses, Short et al. [39] examined the relationship between PTSS and subsequent sleep disturbance (i.e., sleep efficiency, quality, and nightmares) and found elevated PTSS predicted increased nightmares (but not sleep efficiency or quality) the following night. Dietch et al. [37] examined the bidirectional relationship between sleep disturbance (i.e., sleep duration and quality) and PTSS in a sample of World Trade Center responders oversampled for PTSD (*N* = 202; 82.7% male; 19.3% with PTSD) and found reduced sleep duration (but not sleep quality) predicted increased PTSS the following day and increased PTSS predicted reduced sleep duration and quality the following night.

Findings from the prior studies are not consistent and do not provide a clear understanding of the relationship between previous night’s sleep disturbance and next day’s PTSS; Short et al. [38] found sleep efficiency and quality but not duration or nightmares predict PTSS and Dietch et al. [37] found sleep duration but not quality predict PTSS. Thus, additional research is needed to understand the temporal relationship between previous night’s sleep disturbance and PTSS the following day.

The present study used an EMA methodology to examine the relationship between specific sleep characteristics (i.e., previous night’s sleep duration, number of awakenings, sleep quality, trouble falling asleep, and difficulty staying asleep) and PTSS the next day in individuals with probable PTSD. Sleep was assessed once a day and PTSS were assessed four times a day for 15 consecutive days. Use of mixed model analyses (also known as multilevel analyses) allowed us to examine the person’s mean (between-subjects) and last night’s (within-subjects) sleep characteristics associated with PTSS. Use of univariate and multivariate models allowed us to examine the individual and combined associations between sleep characteristics and PTSS. We anticipated that better sleep on any of the sleep measures (i.e., longer sleep duration, fewer awakenings, more highly rated sleep quality, less trouble falling asleep, or less difficulty staying asleep) would be associated with reduced PTSS.

## Methods

### Participants

Current and former U.S. service members (*N* = 141) were recruited from a large military treatment facility. A total of *N* = 249 service members were screened for eligibility to enroll; *n* = 170 screened in and of those *n* = 141 enrolled, *n* = 7 declined enrollment, and *n* = 22 did not return for the enrollment appointment. Of the *N* = 141 who enrolled, *n* = 61 with probable PTSD were included in data analyses, *n* = 59 did not have PTSD, *n* = 12 did not return the daily assessments, *n* = 6 did not provide four or more daily assessments, *n* = 2 did not complete the assessment of probable PTSD, and *n* = 1 was removed as an outlier. This study was part of a larger data collection project looking at post traumatic stress in U.S. military personnel and methods common to the larger study can be found in a prior publication [32].

### Procedure and measures

#### Recruitment and enrollment screening

Advertisements and recruiting personnel stated that the study was seeking to enroll individuals with post traumatic stress symptoms. Service members self-referred for enrollment and completed a 26 symptom screening questionnaire, which included 18 PTSD symptoms from the PTSD Checklist for the Diagnostic and Statistical Manual of Mental Disorders-Fifth Edition (PCL-5; DSM-5) [40], 6 depression symptoms from the Patient Health Questionnaire Depression Scale (PHQ-9) [41, 42], and 2 generalized anxiety symptoms from the Generalized Anxiety Disorder-7 (GAD-7) [43]. The timing of the 26 questions was “…over the past month” and the question response format was 0 (*Not at all*) to 10 (*Extremely*). Individuals who scored 40 or more (out of 0–260 range) were enrolled in the study.

#### Assessment of PTSD

After enrollment, participants completed a questionnaire-based assessment of exposure to traumatic events, which included 79 items that were adapted from multiple sources or developed for use in this study (see Supplement 1). All participants had at least one qualifying traumatic exposure. The 20-item PCL-5 [40] was used to determine whether participants had probable PTSD versus no PTSD. PCL-5 response choices were 0 (*Not at all*) to 4 (*Extremely*) and the symptom severity score range was 0–80. A diagnosis of probable PTSD was made by considering each item rated 2 (*Moderately*) or higher as an endorsed symptom, then following the DSM-5 diagnostic criteria requiring one or more cluster A traumatic exposures, one or more items from clusters B and C, two or more items from clusters D and E, and a symptom severity score of 38 or higher [40]. Participants meeting the probable PTSD criteria (*N* = 61), hereafter referred to as those with PTSD, are the primary focus of this manuscript.

#### Daily assessments

Using an EMA methodology, participants completed four daily assessments per day for the following 15 days (see prior publication [32] for details of daily assessments). The first 19 consecutive subjects (31.1%) completed daily assessments on paper questionnaires and the next 42 subjects (68.9%) completed the same assessments on an Apple Inc. iPad 2 with software designed for this study. Data collection by paper versus electronic assessments was controlled for all analyses and was not a significant covariate. In total, *N* = 2885 daily assessments were collected. Of those, *n* = 78 (2.7%) were dropped from data analysis because they were completed too early (*n* = 13), too late (*n* = 41), or were missing the completion date or time (*n* = 24). Of the *N* = 2807 assessments included in the analyses, *n* = 2095 (74.6%) were completed within 0–2 h, *n* = 512 (18.2%) within 2–4 h, and *n* = 198 (7.1%) within 4–6 h. The overall adherence rate (i.e., percentage completed out of 3660 possible daily assessments) was 76.7% in the present sample. Participants were not compensated for completing assessments.

#### PTSS

Daily PTSS were assessed using 18 PCL-5 items [40], which were included on all four daily assessments. The response format of the PCL-5 items was modified to an 11-point scale, 0 (*Not at all*) to 10 (*Extremely*), with a 0–180 symptom severity score range. The change from a 5-point scale to an 11-point scale was unlikely to affect the mean but was likely to produce data with more variance [44]. Items were also modified to be relevant for repeated assessments. Items in the first daily assessment contained the timing phrase “…since you awakened” and items in the second, third, and fourth daily assessment contained the phrase “…in the last couple of hours.” Item instructions were “Below is a list of problems that people sometimes have in response to very stressful experiences. Please read each problem carefully and then circle one of the numbers on the 0-10 scale where 0 means *Not at all* and 10 means *Extremely* to indicate how much you have been bothered by that problem (…timing phrase here).” Since the population under study may have experienced multiple traumatic events, participants were not required to consider a specific traumatic event when responding to the items (e.g., “Repeated, disturbing, and unwanted memories of a stressful experience.”).

#### Sleep characteristics

Sleep was assessed on the first daily assessment with 23 items (see Supplement 2).

##### Sleep duration

Sleep duration was assessed with one item adapted from the Pittsburgh Sleep Quality Index (PSQI) [45], “How many hours of actual sleep did you get last night? (*This may be different than the number of hours you spent in bed*).” Participants wrote in the number of hours of sleep and, if necessary, responses were rounded to the nearest quarter hour.

##### Number of awakenings

Number of awakenings was assessed with one item, “How many times did you wake up during the night last night?” Participants wrote in the number of awakenings.

##### Sleep quality

Sleep quality was assessed with one item adapted from the PSQI, “How would you rate your sleep quality overall last night?” Response choices ranged from 0 (*Very bad*) to 3 (*Very good*).

##### Sleep problems: trouble falling asleep and difficulty staying asleep

Sleep problems were assessed with 20 items adapted from the PCL-5 [40], PSQI [45], SLEEP-50 [46] or developed for use in this study by the authors (RJU & CSF). Item instructions were “Below is a list of sleep problems. Please fill in the bubble according to what you experienced last night.” and response choices were 0 (*No*) and 1 (*Yes*). For analysis, items were grouped into sleep problem dimensions based on existing sleep scale factors (e.g., sleep disturbances component in the PSQI) and the authors’ (QB, RJU, JW, GHW, & CSF) clinical consensus. Three items formed the dimension measuring trouble falling asleep, eight items formed the dimension measuring somatic disturbance/sleep environment, three items formed the dimension measuring parasomnia, and six items formed the dimension measuring difficulty staying asleep. A confirmatory factor analysis on a larger sample including subjects with and without PTSD supported these dimensions (see Supplement 3). The trouble falling asleep and difficulty staying asleep dimensions include items that are common sleep disturbances for individuals with PTSD [6, 7]. However, the somatic disturbance/sleep environment dimension is conceptually different from other sleep disturbances and the parasomnia dimension was so rarely endorsed that it could not be adequately measured, and both of these dimensions were dropped from further analyses. The Chronbach’s alpha internal reliability of the dimensions, assessed at the person level, was acceptable or good: trouble falling asleep (α = .77) and difficulty staying asleep (α = 0.82). The mean of the items within the trouble falling asleep and difficulty staying asleep dimensions was used in the analyses, indicating the percent of the sleep problem that the participant experienced during the previous night.

### Data analyses

The association between measures of the previous night’s sleep characteristics and PTSS the following day were assessed using linear mixed models with daily assessments (Level 1) nested within subjects (Level 2). Analyses consisted of three steps. The first step was to explore the within-subjects covariance structure. After examining three models, we selected the modified AR(1) model as having the best within-subjects covariance structure [31] (see Supplement 4 for further details).

In step two, we examined the influence of each sleep characteristic in separate mixed models adjusted for gender, age, race, education, and phase of the study. Because the sleep characteristics were time varying variables, each was partitioned into between-subjects (person mean over time; i.e., sleep duration on average) and within-subjects (last night, centered at person mean; i.e., sleep duration last night) level predictors. Also, models were tested with and without a random slope of the last night sleep characteristic. These analyses allowed us to examine the influence of the individuals’ mean across 15 days and the night-to-night variation.

In step three, we included the set of significant sleep variables obtained from the previous step to predict PTSS in a multivariate linear mixed model. Statistical analyses were performed in PC SAS version 9.3 (SAS Institute, Cary, North Carolina).

## Results

### Sample demographics and descriptive statistics

Mean age of participants (*N* = 61) was 38.6 (*SD* = 12.1; range 22–76). Approximately half (54.1%, *n* = 33) were male, the majority were white (63.9%, *n* = 39), and 47.5% (*n* = 29) had a Bachelor’s degree or higher (see Table 1). Mean PTSS score (range 0–180) was 67.1 (*SD* = 39.0). The mean sleep duration per night was 5.3 (*SD* = 2.0), mean number of awakenings was 2.6 (*SD* = 2.1), and mean sleep quality rating was 1.4 (*SD* = 0.8). The mean of trouble falling asleep was 0.53 (*SD* = 0.38) and mean of difficulty staying asleep was 0.36 (*SD* = 0.30).

**Table 1:** Sample Demographics and Descriptive Statistics, *N* = 61

Categorical variable	% (*n*)
Gender	
Male	54.1 (33)
Female	45.9 (28)
Race	
White	63.9 (39)
Others	36.1 (22)
Education	
High school or G.E.D	4.9 (3)
Some college/technical school	47.5 (29)
Bachelor’s degree	21.3 (13)
Graduate degree	26.2 (16)
Marital Status	
Currently married	57.4 (35)
Not currently married	42.6 (26)
Continuous variable	*M* (*SD*)
Age (range = 22–76)	38.6 (12.1)
PTSS (0–180)	67.1 (39.0)
Sleep	
Sleep duration (0–13)	5.3 (2.0)
Number of awakenings (0–16)	2.6 (2.1)
Sleep quality (0–3)	1.4 (0.8)
Trouble falling asleep (0–1)	0.53 (0.38)
Difficulty staying asleep (0–1)	0.36 (0.30)

### Association between PTSS and sleep characteristics

**Fig. 1:**
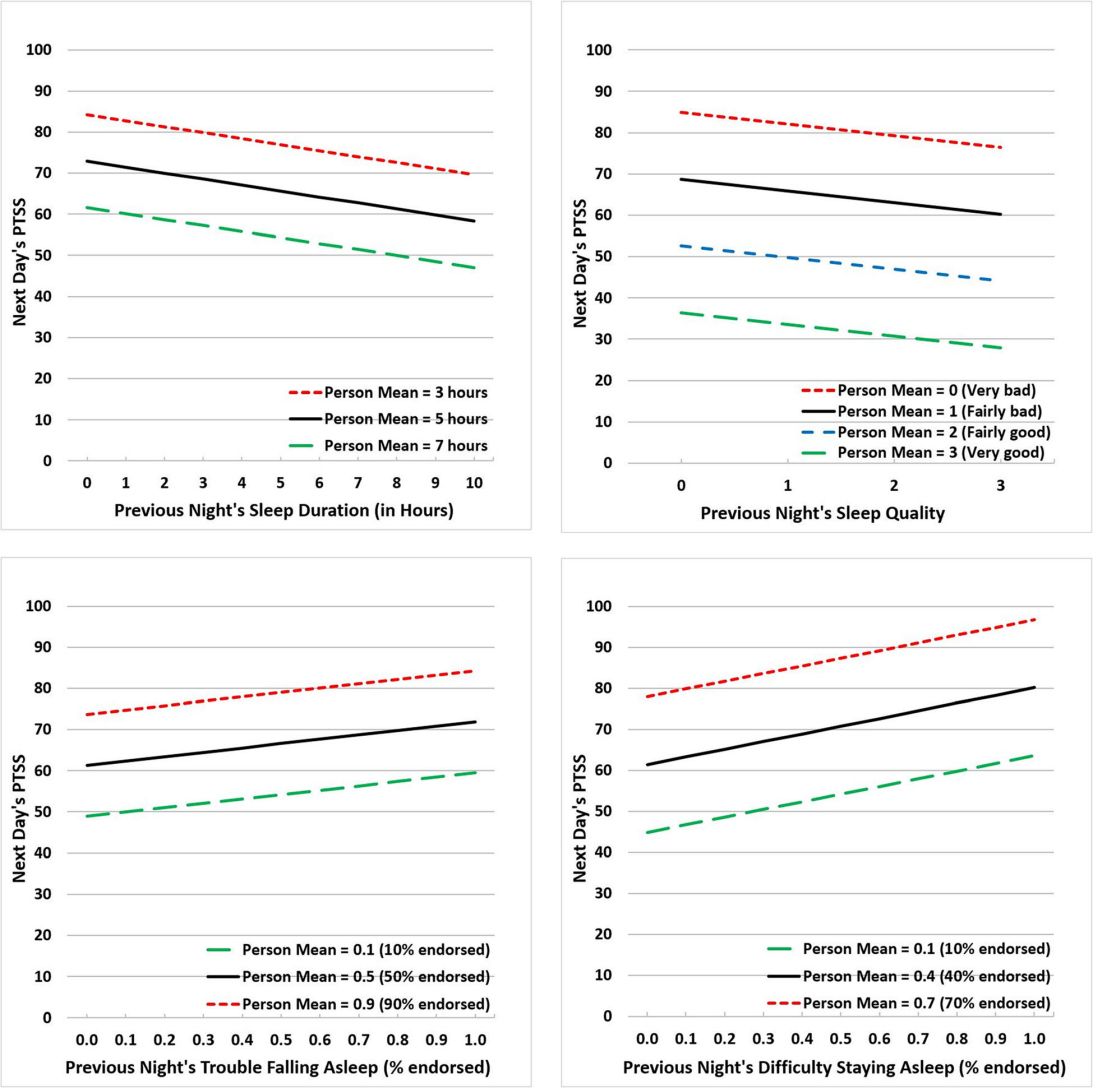
Next Day’s PTSS Predicted by the Previous Night’s Sleep Duration, Sleep Quality, Trouble Falling Asleep, and Difficulty Staying Asleep. *Note*. Least squares means of PTSS, adjusted for demographic characteristics and within-subjects correlations, estimated for each sleep variable with various levels of last night measures, shown as horizontal variables and stratified by different person mean measures. For sleep quality, the four lines of person mean measures reflect the four response options from 0 (*Very bad*) to 3 (*Very good*). For sleep duration, trouble falling asleep, and difficulty staying asleep, the three lines of person mean measures reflect the grand mean and one standard deviation above or below the grand mean. The grand means were 5.3, 1.4, 0.53, and 0.36 for sleep duration, sleep quality, trouble falling asleep, and difficulty staying asleep

We examined each sleep characteristic (sleep duration, number of awakenings, sleep quality, trouble falling asleep, and difficulty staying asleep) separately adjusting for covariates (see Table 2). PTSS was significantly associated with sleep duration (person mean $\hat{\beta}$ = − 7.10, *p* = .063; last night $\hat{\beta}$ = 1.45, *p* < .001), sleep quality (person mean $\hat{\beta}$ = − 18.98, *p* = .109; last night $\hat{\beta}$ = − 2.80, *p* < .001), trouble falling asleep (person mean $\hat{\beta}$ = 41.52, *p* = .006, and last night $\hat{\beta}$ = 10.54, *p* < .001), and difficulty staying asleep (person mean $\hat{\beta}$ = 73.99, *p* = .001, and last night $\hat{\beta}$ = 18.73, *p* < .001). Random slope was significant only for difficulty staying asleep (random slope variance = 310.19, *p* = .005) and the fixed effects (person mean $\hat{\beta}$ = 71.34, *p* = .001; last night $\hat{\beta}$ = 17.69, *p* < .001) were similar to the model without the random slope. The last night measure of sleep duration, sleep quality, trouble falling asleep, and difficulty staying asleep accounted for 7.3, 7.6, 10.7, and 16.5% of the within-subjects variation, respectively. The estimated least square means of PTSS are shown in Fig. 1 by last night measures as horizontal variables, stratified by various levels of person mean measures for the four significant last night predictors. For sleep quality, the four lines of person mean measures reflect the four response options from 0 (*Very bad*) to 3 (*Very good*). For sleep duration, trouble falling asleep, and difficulty staying asleep, the three lines of person mean measures reflect the grand mean and one standard deviation above or below the grand mean. For example, compared to a person who endorsed 40% of the difficulty staying asleep items on average (i.e., person mean = 40%), persons who endorsed 70% of the difficulty staying asleep items (i.e., person mean = 70%) were associated with a 21.20 (30% of $\hat{\beta}$ = 73.99) increase in PTSS, controlling for the last night variable. In addition, the horizontal line represents last night measures. For example, compared to one night where 40% of the difficulty staying asleep items were endorsed, one night where 70% of the difficulty staying asleep items were endorsed was associated with a 5.62 (30% of 18.73) increase in PTSS, controlling for the person level variable.

**Table 2:** Predicting Next Day’s PTSS by Previous Night’s Sleep Characteristics

Sleep characteristics	$\hat{\beta}$	CI	*p*
Univariate sleep characteristics^a^			
Sleep duration			
Person mean	−7.10	[−14.59, 0.390]	.063
Last night^b^	−1.45	[−2.18, −0.73]	<.001
Number of awakenings			
Person mean	1.03	[−4.84, 6.88]	.727
Last night	0.70	[−0.07, 1.47]	.073
Sleep quality			
Person mean	−18.98	[−42.34, 4.38]	.109
Last night	−2.82	[−4.42, −1.23]	<.001
Trouble falling asleep			
Person mean	41.52	[12.35, 70.69]	.006
Last night	10.54	[5.99, 15.09]	<.001
Difficulty staying asleep^c^			
Person mean	73.99	[31.63, 116.35]	.001
Last night	18.73	[13.68, 23.78]	<.001
Multivariate sleep characteristics^d^			
Sleep duration			
Person mean	−2.28	[−9.96, 5.41]	.555
Last night	−0.93	[−1.73, −0.12]	.024
Sleep quality			
Person mean	−6.91	[−29.72, 15.91]	.546
Last night	0.41	[−1.45, 2.27]	.667
Trouble falling asleep			
Person mean	21.74	[−10.51, 54.00]	.182
Last night	5.65	[0.92, 10.39]	.019
Difficulty staying asleep			
Person mean	53.95	[6.98, 100.93]	.025
Last night	16.61	[11.16, 22.06]	<.001

*Note*. ^a^Single variable analysis adjusted for demographic covariates. ^b^The partitioned last night variable was created as the difference between the person mean and the last night. ^c^Random slope was tested for each sleep characteristic and there was a significant random slope of difficulty staying asleep, and the corresponding fixed effects (person mean $\hat{\beta}$ = 71. 34, *p* = .001, and last night $\hat{\beta}$ = 17.69, *p* < .001) were similar to the model without the random slope. ^d^Person mean and last night variables of sleep quality were not statistically significant and were removed from the final model. The model included sleep duration, trouble falling asleep, difficulty staying asleep, and demographic covariates

### Association between PTSS and multiple sleep characteristics

We next examined the association between all statistically significant sleep predictors as multiple predictors and PTSS (see Table 2). Last night measures of sleep duration, trouble falling asleep, and difficulty staying asleep were associated with PTSS (sleep duration $\hat{\beta}$ = − 0.93, *p* = .024; trouble falling asleep $\hat{\beta}$ = 5.65, *p* = .019; and difficulty staying asleep $\hat{\beta}$ = 16.61, *p* < .001). The person mean of difficulty staying asleep was associated with a 53.95 increase in PTSS (*p* = .025). Controlling for other sleep variables in the model, the person mean and last night variables of sleep quality (person mean $\hat{\beta}$ = − 6.91, *p* = .546; last night $\hat{\beta}$ = 0.41, *p* = .667) were no longer statistically significant.

Results for the final model, including sleep duration, trouble falling asleep, and difficulty staying asleep, are reported in Supplement 5. Last night measures of all three sleep variables were associated with PTSS (sleep duration $\hat{\beta}$ = − 0.85, *p* = .018; trouble falling asleep $\hat{\beta}$ = 5.52, *p* = .021; and difficulty staying asleep $\hat{\beta}$ = 16.30, *p* < .001). In addition, the person mean of difficulty staying asleep was associated with a 56.24 increase in PTSS (*p* = .018). All three sleep variables together explained 18.6% of the within-subjects, state-level systematic variation (216.00 compared to 265.44 in the model with covariates only) and 20.7% of the between-subjects variation (956.55 compared to 1205.61 in the model with covariates only) in PTSS. Adding random slopes of difficulty staying asleep resulted in similar fixed effects for all predictors.

## Discussion

Using an EMA methodology, this study examined the relationship between sleep characteristics (i.e., previous night’s sleep duration, number of awakenings, sleep quality, trouble falling asleep, and difficulty staying asleep) and PTSS the next day in individuals with probable PTSD. Our findings compare well and expand upon previous research through use of a large sample with PTSD, thorough assessment of PTSS, and strong methodology. We found that individuals’ previous night (within-subjects) and person mean (between-subjects) sleep characteristics were significantly associated with PTSS. Specifically, shorter sleep duration, more trouble falling asleep, and more difficulty staying asleep the previous night predicted higher PTSS the following day and more difficulty staying asleep on average predicted higher PTSS.

Our initial analyses examined each sleep characteristic in a separate single predictor model similar to those used by Short et al. [38] and Dietch et al. [37]. We found previous night’s sleep duration, sleep quality, trouble falling asleep, and difficulty staying asleep predicted next day PTSS. In comparison, Short et al. found previous night’s sleep quality and sleep efficiency, which includes sleep onset latency (i.e., trouble falling asleep) and wake after sleep onset (i.e., difficulty staying asleep), but not sleep duration, predicted next day PTSS. Consistent with our findings but in contrast to Short et al., Dietch et al. found previous night’s sleep duration, but not sleep quality, predicted next day PTSS. Differences in findings may be due to the number and diagnostic status of subjects, PTSS assessment methods, and sleep measures. Our sample included 61 current and former service members with PTSD, Short et al. included 30 community and university research pool subjects with PTSD, and Dietch et al. included 202 disaster responders of which 39 (19.3%) had PTSD. Dietch et al.’s sample may have had fewer sleep disturbances as individuals without PTSD are less likely to have sleep disturbances than those with PTSD [3]. Our daily PTSS assessments were more comprehensive; we used all 18 non-sleep items of the PCL-5 four times daily for 15 days while Short et al. used 10 PCL-5 items four times daily for 8 days and Dietch et al. used 8 PCL-5 items three times daily for 7 days. Further, our study and Short et al.’s study used one item from the PSQI [45] to measure sleep quality and both studies found an association between sleep quality and PTSS whereas Dietch et al. used a different measure and did not find such an association. Despite study differences, there was consistency in finding that previous night’s sleep duration and sleep quality predict next day PTSS in single predictor models.

In contrast to Short et al. and Dietch et al., we also examined person mean (between subjects). Individuals differ in their average amount of sleep per day and looking at averages may be important to understanding the relationship between sleep and PTSS. Trouble falling asleep and difficulty staying asleep significantly predicted PTSS, which indicates that individuals who average somewhat less trouble falling asleep and less difficulty staying asleep will average lower PTSS.

Importantly, because sleep characteristics overlap, we examined our findings in a multivariate model to identify unique contributions to PTSS of significantly associated sleep characteristics. Previous night’s (within subjects) sleep duration, trouble falling asleep, and difficulty staying asleep continued to predict next day PTSS, but sleep quality did not. This suggests that sleep quality, which we measured with one item adapted from the PSQI [45], is a broad index of sleep that may include individuals’ perceptions of sleep duration, trouble falling asleep, and difficulty staying asleep. This is partially consistent with research finding that sleep quality as measured by the full PSQI is a multidimensional construct where the factor of perceived sleep quality (i.e., PSQI subscales: subjective sleep quality, sleep latency, sleep disturbance, and daytime dysfunction) is associated with PTSD and the factor of efficiency/duration (i.e., PSQI subscales: sleep duration and habitual sleep efficiency) is not associated with PTSD [47]. Further research is needed to understand the conceptual elements of sleep quality. In addition, in the multivariate model the person mean of difficulty staying asleep significantly continued to predict PTSS indicating that individuals who average somewhat less difficulty staying asleep will average lower PTSS.

As sleep disturbance is an independent risk factor for PTSD that exacerbates and maintains PTSD and may remain after treatment for PTSD, greater emphasis on early assessment, diagnosis, and treatment of sleep problems is warranted. Often, sleep becomes the target of intervention only after PTSD treatment has had limited efficacy. The present study and others [48] suggest the importance of examining treatment for sleep prior to and simultaneous with treatment for PTSD. Sleep affects cognitive functioning independently of PTSD [49] and poor sleep has been associated with a range of cognitive impairments including impairment of memory [50, 51]. PTSD treatments require effective advanced learning and memory functions [28]. Early interventions for sleep may offer less mental health stigma compared to treatment for PTSD, reduce daily symptoms, and build therapeutic rapport and overall stability prior to engaging in more challenging trauma-focused therapies [52]. Further studies are needed to determine the optimal temporal relationship between sleep and PTSD treatments.

Our study identified associative relationships between sleep characteristics and PTSS. Experimental studies that identify causal relationships are needed and both short-term interventions that improve night-to-night sleep and longer-term interventions to improve an individual’s average sleep should be studied. It is important to understand the different sleep characteristics and each characteristic’s relationship to PTSS to better target clinical interventions to have a substantial impact on symptoms.

There are several limitations of the present study. Measures of sleep and PTSS were obtained by self-report and such measures may be less accurate compared to objective measures of sleep or a psychiatric assessment by a clinician. This limitation is partially mitigated by use of well-validated self-assessment measures. Self-report data are vulnerable to recall and response bias. However, the majority of the daily assessments were collected at the time of the behavior of interest or within hours afterwards. It is possible that when reporting sleep events a participant may have endorsed more than one item within a sleep dimension. Since we did not track participants’ daily schedule, we do not know to what extent the trends in sleep and PTSS vary with work or other activities. Information on sleep apnea was not collected and we cannot rule out its presence in this sample. This is a descriptive study and experimental studies are needed to clarify causal relationships. Lastly, because our sample consisted only of current and former service members, our findings may not generalize to civilians.

In order to better understand the association between sleep characteristics and PTSS, future studies should explore the mechanisms by which the previous night’s sleep disturbance contributes to the next day’s variation in PTSS. These may include symptoms of depression, neurobiological processes, alterations in sleep architecture, and the effects of traumatic event cues, interpersonal conflicts, or other stressful events at work or home. For example, negative affect was found to mediate the relationship between the previous night’s sleep quality and the next day’s PTSS [38]. Future research may determine whether sleep characteristics have a greater impact on specific symptoms (e.g., hyperarousal symptoms) versus other symptoms. Some sleep characteristics such as sleep duration may vary during the week and it will be important to examine whether they influence the day of week variation in PTSS [32]. Finally, future studies should examine whether interventions for sleep disturbance can prevent the onset of PTSD.

The findings of our study suggest that sleep duration, trouble falling asleep, and difficulty staying asleep vary night-to-night with subsequent day-to-day changes in PTSS. Because sleep problems are treatable, these findings raise the possibility that improving sleep may be a means to reduce PTSS in individuals with PTSD. Importantly, each of these sleep characteristics independently predicts next day PTSS even when adjusted for each other. Further study of sleep disturbance and its relationship to PTSS may add to the understanding of PTSD and to identifying modifiable precipitating factors for sleep disturbance and PTSD.

## Conclusions

This study examined the relationship between sleep characteristics and PTSS in individuals with probable PTSD. Both previous night (within-subjects) and person mean (between-subjects) sleep characteristics were significantly associated with PTSS. In separate single predictor models, previous night’s sleep duration, sleep quality, trouble falling asleep, and difficulty staying asleep predicted next day PTSS. In a multivariate model which identified the unique contributions of each sleep characteristic, previous night’s sleep duration, trouble falling asleep, and difficulty staying asleep continued to predict next day PTSS. In addition, the person mean of difficulty staying asleep significantly predicted PTSS. Our study identified associative relationships between sleep characteristics and PTSS, and experimental studies that identify causal relationships are needed. These findings raise the possibility that improving sleep may be a means to reduce PTSS in individuals with PTSD.

## Supplementary information

**Additional file 1: Supplement 1.** Assessment of Traumatic Event Exposure. **Supplement 2.** Sleep Items on the First Daily Assessment. **Supplement 3.** Description of the Confirmatory Factor Analysis and Larger Sample. **Supplement 4a.** Comparison of Within-Subjects Covariance Structure. **Supplement 4b.** Table of Model Specification on Within-Subjects Residuals and Decomposition of Variance. **Supplement 5.** Final Model Predicting PTSS by Multiple Sleep Variables.

## Data Availability

The datasets used and/or analyzed during the current study are available from the corresponding author on reasonable request.

## Abbreviations

AR(1): Autoregression assumption
CFA: Confirmatory factor analysis
CS: Compound symmetry
DSM-5: Diagnostic and Statistical Manual of Mental Disorders-Fifth Edition
EMA: Ecological momentary assessment
GAD-7: Generalized Anxiety Disorder 7
PCL-5: PTSD Checklist for the DSM-5
PHQ-9: Patient Health Questionnaire Depression Scale
PSQI: Pittsburg Sleep Quality Index
PTSD: Post traumatic stress disorder
PTSS: Post traumatic stress symptoms
