# Supplementary material for: Post traumatic stress symptom variation associated with sleep characteristics
Source: BMC Psychiatry. 2020 Apr 16;20:174. doi: 10.1186/s12888-020-02550-y (PMC7164146; doi:10.1186/s12888-020-02550-y)
Supplement: Supplementary file 1 — Additional file 1: Supplement 1. Assessment of Traumatic Event Exposure. Supplement 2. Sleep Items on the First Daily Assessment. Supplement 3. Description of the Confirmatory Factor Analysis and Larger Sample. Supplement 4a. Comparison of Within-Subjects Covariance Structure. Supplement 4b. Table of Model Specification on Within-Subjects Residuals and Decomposition of Variance. Supplement 5. Final Model Predicting PTSS by Multiple Sleep Variables. [file 12888_2020_2550_MOESM1_ESM.docx]

**Supplement 1.** Assessment of Traumatic Event Exposure

Exposure to a traumatic event was assessed with an aggregate of 79 items adapted from multiple sources or developed for use in this study. Response choices varied by item but any response indicating that a traumatic event had occurred at least once in the individual’s life was considered endorsement of that item.

Traumatic Event Exposure Items

| Childhood Experiences | |
| --- | --- |
| 1. | You were beaten up, had things stolen from you, or were terrorized by bullies at school or in the neighborhood^a^ |
| 2. | You were physically abused at home^a,b^ |
| 3. | You were sexually abused at home^a,b^ |
| 4. | Someone touched you or made you touch them in a sexual way against your will^a,b^ |
| 5. | Someone in your family hit you so hard that it left bruises or marks^a,b^ |
| 6. | Your parents (or the people who raised you) hit each other or beat each other up^a,c^ |
| 7. | You witnessed or experienced a violent crime, like a shooting or a rape^d^ |
|  |  |
| PTSD Diagnosis Criterion A Stressful Experiences | |
| 8. | Have you ever been in danger of being killed or injured prior to being in the military^e^ |
| 9. | Have you ever been in danger of being killed or injured during military service^e^ |
| 10. | Have you ever been in danger of being killed or injured since military service^e^ |
| 11. | Have you ever witnessed someone in danger of being killed or injured prior to being in the military^e^ |
| 12. | Have you ever witnessed someone in danger of being killed or injured during military service^e^ |
| 13. | Have you ever witnessed someone in danger of being killed or injured since military service^e^ |
|  |  |
| Life Events Checklist (Endorsed if response was *Happened to me* or *Witnessed it*) | |
| 14. | Natural disaster (for example, flood, hurricane, tornado, earthquake)^f,g^ |
| 15. | Fire or explosion^f,g^ |
| 16. | Transportation accident (for example, car accident, boat accident, train wreck, plane crash)^f,g^ |
| 17. | Serious accident at work, home, or during recreational activity^f,g^ |
| 18. | Exposure to toxic substance (for example, dangerous chemicals, radiation)^f,g^ |
| 19. | Physical assault (for example, being attacked, hit, slapped, kicked, beaten up)^f,g^ |
| 20. | Assault with a weapon (for example, being shot, stabbed, threatened with a knife, gun, bomb)^f,g^ |
| 21. | Sexual assault (rape, attempted rape, made to perform any type of sexual act through force or threat of harm)^f,g^ |
| 22. | Other unwanted or uncomfortable sexual experience^f,g^ |
| 23. | Combat or exposure to a war-zone (in the military or as a civilian)^f,g^ |
| 24. | Captivity (for example, being kidnapped, abducted, held hostage, prisoner of war)^f,g^ |
| 25. | Life-threatening illness or injury^f,g^ |
| 26. | Severe human suffering^f,g^ |
| 27. | Sudden violent death (for example, homicide, suicide)^f,g^ |
| 28. | Sudden accidental death^g^ |
| 29. | Serious injury, harm, or death you caused to someone else^f,g^ |
|  |  |
| Combat Experiences Scale | |
| 30. | Having to aid in the removal of unexploded land mines^h^ |
| 31. | Improvised explosive device/booby trap exploded near you^h^ |
| 32. | Patrolling areas (or riding in areas) where there were land mines^h^ |
| 33. | Being attacked or ambushed^h^ |
| 34. | Clearing/searching homes or buildings^h^ |
| 35. | Receiving small arms fire^h^ |
| 36. | Seeing dead bodies or body parts^h^ |
| 37. | Shooting or directing fire at the enemy^h^ |
| 38. | Being in threatening situations where you were unable to respond because of rules of engagement^h^ |
| 39. | Witnessing a friendly fire incident^h^ |
| 40. | Had a close call, dud landed near you^h^ |
| 41. | Provided aid to the wounded^h^ |
| 42. | Saved the life of a soldier or civilian^h^ |
| 43. | Handling or uncovering dead bodies or body parts^h^ |
| 44. | Witnessing violence within the local population or between ethnic groups^h^ |
| 45. | Witnessing an accident which resulted in serious injury or death^h^ |
| 46. | Seeing dead or seriously injured Americans^h^ |
| 47. | Had a close call, was shot or hit but protective gear saved you^h^ |
| 48. | Being wounded^h^ |
| 49. | Had a buddy shot or hit who was near you^h^ |
| 50. | Knowing someone seriously injured or killed^h^ |
| 51. | Having a member of your own unit become a casualty^h^ |
| 52. | Receiving incoming artillery rocket or mortar fire^h^ |
| 53. | Being directly responsible for the death of an enemy combatant^h^ |
| 54. | Being directly responsible for the death of a non-combatant^h^ |
| 55. | Successfully engaged the enemy^h^ |
| 56. | Being directly responsible for the death of a US or ally personnel^h^ |
| 57. | Calling in fire on the enemy^h^ |
| 58. | Engaging in hand to hand combat^h^ |
| 59. | Witnessing brutality/mistreatment toward non-combatants^h^ |
| 60. | Clearing/searching caves or bunkers^h^ |
|  |  |
| Deployment Experiences | |
| 61. | Being in an accident^i^ |
| 62. | Witnessing a suicide bombing^i^ |
| 63. | Having contact with traumatized civilians^i^ |
| 64. | Being shot at^i^ |
| 65. | During your most recent deployment, how often did you think you were in danger of being injured or killed^i^ |
| 66. | During your most recent deployment, did you handle any dead bodies^i^ |
|  |  |
| Life Experiences | |
| 67. | Serious physical assault (e.g. mugging), sexual assault, or rape^j,k^ |
| 68. | Serious assault happened to a close friend or relative^j,k^ |
| 69. | Murder of a close friend or relative^j,k^ |
| 70. | Suicide of a close friend or relative^j,k^ |
| 71. | Attempted suicide of a close friend or relative^j,k^ |
| 72. | Combat death of a close friend or relative^j,l^ |
| 73. | Accidental death of a close friend or relative^j,k^ |
| 74. | You witnessed someone being seriously injured or killed^j,k,l^ |
| 75. | You discovered or handled a dead body^j,k^ |
| 76. | You had a life-threatening illness or injury^j,l^ |
| 77. | You were in a disaster (e.g., hurricane, fire, flood, earthquake) where you could have died^j,k^ |
| 78. | You had any other experience that put you at risk of death or serious injury^j,k,l^ |
| 79. | You had a close loved one who had an experience that put them at risk of death or serious injury^j,k^ |
|  |  |
| Source | |
| ^a^ | Army STARRS New Soldier Study Questionnaire [1, 2] Available at: www.starrs-ls.org |
| ^b^ | Childhood Trauma Questionnaire (CTQ) [3, 4, 5] |
| ^c^ | Adverse Childhood Experiences (ACE) [6, 7] |
| ^d^ | Semi-Structured Assessment for Drug Dependence and Alcoholism (SSADDA) [8] |
| ^e^ | Posttraumatic Stress Disorder, diagnostic criterion A [9, 10] |
| ^f^ | Life Events Checklist (LEC) [11] |
| ^g^ | Life Events Checklist for DSM-5 (LEC-5) [12] |
| ^h^ | Combat Exposure Scale (CES) [13] |
| ^i^ | Developed for use in this study by the authors (QMB, RJU, CSF) |
| ^j^ | Army STARRS All Army Study Questionnaire [1, 2] Available at: www.starrs-ls.org |
| ^k^ | Deployment Risk and Resilience Inventory (DDRI) [14, 15] |
| ^l^ | Joint Mental Health Advisory Team 7 (J-MHAT 7) Operation Enduring Freedom 2010 Afghanistan |

**References**

1 Kessler, R. C., Colpe, L. J., Fullerton, C. S., Gebler, N., Naifeh, J. A., Nock, M. K.,…Heeringa, S. G. (2013). Design of the Army Study to Assess Risk and Resilience in Service members (Army STARRS). *International Journal of Methods in Psychiatric Research, 22*(4), 267-275. doi: 10.1002/mpr.1401

2 Ursano, R. J., Colpe, L. J., Heeringa, S. G., Kessler, R. C., Schoenbaum, M., & Stein, M. B. (2014). The Army Study to Assess Risk and Resilience in Servicemembers (Army STARRS). *Psychiatry: Interpersonal and Biological Processes, 72*(2), 107-119. doi:10.1521/psyc.2014.77.2.107

3 Bernstein, D. P., Ahluvalia, T., Pogge, D., & Handelsman, L. (1997). Validity of the Childhood Trauma Questionnaire in and adolescent psychiatric population. *Journal of the American Academy of Child & Adolescent Psychiatry, 36*(3), 340-348. doi: 10.1097/00004583-199703000-00012

4 Bernstein, D. P., Fink, F., Handelsman, L., Foote, J., Lovejoy, M., Wenzel, K., … Ruggiero, J. (1994). Initial reliability and validity of a new retrospective measure of child abuse and neglect. *American Journal of Psychiatry, 151*(8), 1132-1136. doi: 10.1176/ajp.151.8.1132

5 Bernstein, D. P., Stein, J. A., Newcomb, M. D., Walker, E., Pogge, D., Ahluvalia, T., … Zule, W. (2003). Development and validation of a brief screening version of the Childhood Trauma Questionnaire. *Child Abuse & Neglect, 27*(2), 169-190. doi: 10.1016/S0145-2134(02)00541-0

6 Anda, R. F., Felitti, V. J., Bremner, J. D., Walker, J. D., Whitfield, C., Perry, B. D., … Giles, W. H. (2006). The enduring effects of abuse and related adverse experiences in childhood. A convergence of evidence from neurobiology and epidemiology. *European Archives of Psychiatry and Clinical Neuroscience, 256*(3), 174-186. doi: 10.1007/s00406-005-0624-4

7 Felitti, V. J., Anda, R. F., Nordenberg, D., Williamson, D. F., Spitz, A. M., Edwards, V., … Marks, J. S. (1998). Relationship of childhood abuse and household dysfunction to many of the leading causes of death in adults. The Adverse Childhood Experiences (ACE) Study. *American Journal of Preventive Medicine, 14*(4), 245-258. doi: 10.1016/S0749-3797(98)00017-8

8 Pierucci-Lagha, A., Gelernter, J., Feinn R., Cubells, J. F., Pearson, D., Pollastri, A., … Kranzler, H. R. (2005). Diagnostic reliability of the Semi-structured Assessment for Drug Dependence and Alcoholism (SSADDA). *Drug and Alcohol Dependence, 80*(3)*,* 303-312. doi: 10.1016/j.drugalcdep.2005.04.005

9 American Psychiatric Association. (2013). *Diagnostic and statistical manual of mental disorders* (5th ed.). Arlington, VA: Author.

10 American Psychiatric Association. (1994). *Diagnostic and statistical manual of mental disorders* (4th ed.). Washington, DC: Author.

11 Gray, M. J., Litz, B. T., Hsu, J. L., & Lombardo, T. W. (2004). Psychometric properties of the Life Events Checklist. *Assessment, 11*(4), 330-341. doi: 10.1177/1073191104269954

12 Weathers, F. W., Blake, D. D., Schnurr, P. P., Kaloupek, D. G., Marx, B. P., & Keane, T. M. (2013). The Life Events Checklist for DSM-5 (LEC-5). Instrument available from the National Center for PTSD at www.ptsd.va.gov

13 Killgore, W. D. S., Cotting, D. I., Thomas, J. L., Cox, A. L., McGurk, D., Vo, A. H., … Hoge, C. W. (2008). Post-combat invincibility: Violent combat experiences are associated with increased risk-taking propensity following deployment. *Journal of Psychiatric Research, 42*, 1112-1121. doi: 10.1016/jpsychires.2008.01.001

14 King, L. A., King, D. W., Vogt, D. S., Knight, J., & Samper, R. E. (2006). Deployment Risk and Resilience Inventory: A collection of measures for studying deployment-related experiences of military personnel and veterans. *Military Psychology, 18*(2), 89-120. doi: 10.1207/s15327876mp1802_1

15 Vogt, D. S., Proctor, S. P., King, D. W., King, L. A., & Vasterling, J. J. (2008). Validation of scales from the Deployment Risk and Resiliency Inventory in a sample of Operation Iraqi Freedom Veterans. *Assessment, 15*(4), 391-403. doi: 10.1177/1073191108316030

**Supplement 2.** Sleep Items on the First Daily Assessment

How many hours of **actual sleep** did you get **last night**? (*This may be different than the number of hours you spent in bed.)* _______

How would you rate your sleep quality overall **last night**?

a. Very bad

b. Fairly bad

c. Fairly good

d. Very good

How many times did you wake up during the night **last night**? _______

Below is a list of sleep problems. Please fill in the bubble according to what you experienced **last night**.

|  | **No** | **Yes** |
| --- | --- | --- |
| *Trouble falling asleep* |  |  |
| Trouble falling asleep^a,b,c^ | ○ | ○ |
| Worried about being able to fall asleep^d^ | ○ | ○ |
| Worried about having disturbing dreams^d^ | ○ | ○ |
| *Somatic disturbance/sleep environment* |  |  |
| Had pain^b^ | ○ | ○ |
| Could not breathe comfortably^b^ | ○ | ○ |
| Coughed or snored loudly^b^ | ○ | ○ |
| Had to get up to use the bathroom^b^ | ○ | ○ |
| Felt too cold^b^ | ○ | ○ |
| Felt too hot^b^ | ○ | ○ |
| It was too noisy in my bedroom (i.e. partner or family member snored)^c^ | ○ | ○ |
| Had to get up because I was hungry or thirsty^d^ | ○ | ○ |
| *Parasomnia* |  |  |
| Performed an action during the night I do not remember (i.e., sleep walking)^c^ | ○ | ○ |
| Saw dreamlike images when falling asleep or waking up^c^ | ○ | ○ |
| Could not move when falling asleep or waking up (i.e., paralyzed)^c^ | ○ | ○ |
| *Difficulty staying asleep* |  |  |
| Trouble staying asleep^a,b^ | ○ | ○ |
| Repeated, disturbing dreams of a stressful experience^a^ | ○ | ○ |
| Had bad or frightening dreams^c^ | ○ | ○ |
| Distressing dreams that did **not** wake me up^d^ | ○ | ○ |
| Woke to a feeling of fear or terror^d^ | ○ | ○ |
| Woke up screaming or in a panic^d^ | ○ | ○ |

*Note*. Sleep items were adapted from the following sources: ^a^PTSD Checklist for the DSM-5 (PCL-5) [1], ^b^Pittsburgh Sleep Quality Index (PSQI) [2], ^c^SLEEP-50 [3], or ^d^developed for use in this study by the authors (RJU & CSF).

**References**

1 Weathers, F. W., Litz, B.T., Keane, T. M., Palmieri, P. A., Marx, B. P., & Schnurr, P. P. (2013). The PTSD Checklist for DSM-5 (PCL-5). Scale available from the National Center for PTSD at ww.ptsd.va.gov.

2 Buysse, D. J., Reynolds, 3rd C. F., Monk, T. H., Berman, S. R., & Kupfer, D. J. (1989). The Pittsburgh Sleep Quality Index: a new instrument for psychiatric practice and research. *Psychiatry Research, 28*, 193-213. doi: 10.1016/0165-1781(89)90047-4

3 Spoormaker, V. I., Verbeek, I., van den Bout, J., & Klip, E. C. (2005). Initial validation of the SLEEP-50 questionnaire. *Behavioral Sleep Medicine, 3*(4), 227-246. doi: 10.1207/s15402010bsm0304_4

**Supplement 3.** Description of the Confirmatory Factor Analysis and Larger Sample

In order to understand the cohesiveness of the sleep problem items, we did a confirmatory factor analysis (CFA) on our four sleep dimensions: trouble falling asleep, somatic disturbance/sleep environment, parasomnia, and difficulty staying asleep. Individual items were grouped by a priori hypotheses about their factor structure prior to the CFA analysis [1]. To fulfill the sample size required for a CFA, we included subjects with and without PTSD from the larger data collection project. Subjects without PTSD underwent the same recruitment, screening, enrollment, and study procedures and as those with PTSD. In the larger data collection project, *N* = 249 current and former service members were screened for eligibility to enroll. A total of *n* = 170 screened in and of those *n* = 141 enrolled, *n* = 7 declined enrollment, and *n* = 22 did not return for the enrollment appointment. Of the *N* = 141 who enrolled, *n* = 120 (*n* = 61 with PTSD and *n* = 59 without PTSD) were included in the CFA analysis, *n* = 12 did not return the daily assessments, *n* = 6 did not provide four or more daily assessments, *n* = 2 did not complete the assessment of probable PTSD, and *n* = 1 was removed as an outlier due to extremely high PTSS scores but failure to meet probable PTSD diagnostic criteria. CFA results indicated that the model with the four sleep problem dimensions had a good fit (χ^2^ = 171.8, *df* = 164, *p* = .322, CFI = .985, TLI = .983, RMSEA = .020, and 90% CI [0.00, 0.047]) [2].

**References**

1 Hurley, A. E., Scandura, T. A., Schriesheim, C. A., Brannick, M. T., Seers, A., Vandenberg, R. J., & Williams, L. J. (1997). Exploratory and confirmatory factor analysis: guidelines, issues, and alternatives. *Journal of Organizational Behavior, 18*, 667-683. doi: 10.1002/(SICI)1099-1379(199711)18:6<667::AID-JOB874>3.0.CO;2-T

2 Kline, R. B. (2016). *Principles and Practice of Structural Equation Modeling*. Guilford Press, New York.

**Supplement 4a.** Comparison of Within-Subjects Covariance Structure

Covariance structure models were compared in order to determine the best within-subjects covariance structure for use in mixed model analyses. Specifically, compound symmetry (CS) was tested against the first-order autoregression assumption (AR[1]) and a modified AR(1) model that considered random measurement error for each assessment, decomposing the error term into two parts: systematic variance by autocorrelation and random measurement error variance [1]. The model with AR(1) covariance structure provided a better fit to the data when compared to a CS structure (Change in -2 log likelihood [-2LL], -2LL = 25325.0 - 24912.9 = 412.1, *df* =1, *p* < .001; see Supplement 4b). The model with the modified AR(1) error structure further improved model fit (Change in -2LL = 89.6, *df* = 1, *p* < .001). The modified AR(1) model was selected for use in analyses.

Taken from the model with the modified AR(1) specification, the estimated mean PTSS total score for a typical participant was 68.87. There were substantial individual differences in person mean total scores (between-subjects variance = 1140.9), “trait-like” differences that consisted of 69.7% of the total variance in daily PTSS reports (ICC = 0.70). The remaining 30.3% was due to fluctuations in PTSS total scores within subjects. The modified AR(1) specification further decomposed the within-subjects fluctuations into a systematic “state-level” component (16.2%, *p* < 0.001), which implies quite high serial autocorrelation (4-hour correlation = 0.85), and a random measurement error component (14.1%, *p* < 0.001).

**References**

1 Schwartz, J. E., & Stone, A. A. (2007). The analysis of real-time momentary data: A practical guide. In A. A. Stone, S. Shiffman, A. A. Atienza, & L. Nebeling (Eds.), *The science of real-time data capture: self-reports in health research* (pp. 76-113). New York, NY: Oxford University Press, Inc.

**Supplement 4b.** Table of Model Specification on Within-Subjects Residuals and Decomposition of Variance

|  | Within-subjects covariance structure | | |
| --- | --- | --- | --- |
| Source of variance | CS^a^ | AR(1)^b^ | Modified AR(1)^c^ |
| Between-subjects | 1172.37 | 1167.25 | 1140.91 |
| Within-subjects | 453.06 | 476.05 | 496.33 |
| Autocorrelated |  | 476.05 | 265.71 |
| Measurement error |  |  | 230.62 |
|  |  |  |  |
| Decomposition of variance (trait-state-error) |  |  |  |
| “Trait-like” (ICC)^d^ | 0.72 | 0.71 | 0.70 |
| “State” (autocorrelation) |  | 0.29 | 0.16 |
| “Error” | 0.28 |  | 0.14 |
| Model fit statistics |  |  |  |
| -2 log likelihood | 25325.0 | 24912.9 | 24823.3 |
| Number of parameters | 2 | 3 | 4 |
| AIC (smaller is better) | 25329.0 | 24918.9 | 24831.3 |
| BIC (smaller is better) | 25333.3 | 24925.2 | 24839.7 |

*Note*. ^a^CS = Compound symmetry. The CS structure assumes the correlation between two assessments is constant regardless of their distance in time. The estimated correlation is calculated by CS/(CS+Residual) = 0.72; ^b^AR(1) = First-order Autoregressive. The AR(1) structure assumes that the correlation between two assessments decreases exponentially as a function of the time interval between them. ^c^The modified AR(1) assumes first-order autoregressively correlated residuals with uncorrelated “measurement error.” ^d^ICC = Intraclass correlation is calculated by the ratio of between-subjects variance by the total variance.

**Supplement 5.** Final Model Predicting PTSS by Multiple Sleep Variables

|  | Multiple sleep variables | | |
| --- | --- | --- | --- |
| Parameter | Estimate | CI | *p* |
| Fixed effects |  |  |  |
| Intercept | 71.48 | [54.04, 88.92] | <.001 |
| Female vs. male | -13.44 | [-30.55, 3.67] | .121 |
| Age (centered at 37.2) | 0.40 | [-0.39, 1.20] | .316 |
| Non-white vs. white | -3.60 | [-23.51, 16.31] | .718 |
| Lower than college vs.  college or higher^a^ | 3.51 | [-17.09, 24.11] | .734 |
| Phase 1 vs. 2 | 6.46 | [-12.53, 25.44] | .498 |
| Sleep duration  Person mean | -2.80 | [-10.24, 4.65] | .454 |
| Last night^b^ | -0.85 | [-1.58, -0.13] | .021 |
| Trouble falling asleep  Person mean | 22.10 | [-9.92, 54.13] | .172 |
| Last night | 5.52 | [0.84, 10.20] | .021 |
| Difficulty staying asleep  Person mean | 56.24 | [10.18, 102.30] | .017 |
| Last night | 16.30 | [11.05, 21.56] | <.001 |
|  |  |  |  |
| Random effects |  |  |  |
| Between-subjects variance | 956.55 |  | <.001 |
| Autocorrelation | 0.86 |  | <.001 |
| Within-subjects systematic variance^c^ | 216.00 |  | <.001 |
| Measurement error | 239.57 |  | <.001 |
| Intraclass correlation^d^ | 0.68 |  |  |

*Note*. ^a^Some college/technical school or higher was set as the reference. ^b^The partitioned last night variable was created as the difference between the person mean and the last night. ^c^The modified AR(1) specification decomposed the within-subjects variation into a systematic “state-level” component (216.0, 15.3%) with high serial autocorrelation (4-hour correlation = 0.86) and a random measurement error component (239.6, 17.0%). ^d^Intraclass Correlation was calculated by the ratio of between-subjects variance and the total variance.
